# P2X_4_ Assembles with P2X_7_ and Pannexin-1 in Gingival Epithelial Cells and Modulates ATP-induced Reactive Oxygen Species Production and Inflammasome Activation

**DOI:** 10.1371/journal.pone.0070210

**Published:** 2013-07-25

**Authors:** Shu-Chen Hung, Chul Hee Choi, Najwane Said-Sadier, Larry Johnson, Kalina Rosenova Atanasova, Hanen Sellami, Özlem Yilmaz, David M. Ojcius

**Affiliations:** 1 Department of Molecular Cell Biology, University of California Merced, Merced, California, United States of America; 2 Health Sciences Research Institute, University of California Merced, Merced, California, United States of America; 3 Department of Periodontology, University of Florida, Gainesville, Florida, United States of America; 4 Emerging Pathogens Institute, University of Florida, Gainesville, Florida, United States of America; 5 Department of Microbiology, Habib Bourguiba University Hospital, Medical School of Sfax, University of Sfax, Sfax, Tunisia; Shanghai Medical College, Fudan University, China

## Abstract

We have previously reported that *Porphyromonas gingivalis* infection of gingival epithelial cells (GEC) requires an exogenous danger signal such as ATP to activate an inflammasome and caspase-1, thereby inducing secretion of interleukin (IL)-1β. Stimulation with extracellular ATP also stimulates production of reactive oxygen species (ROS) in GEC. However, the mechanism by which ROS is generated in response to ATP, and the role that different purinergic receptors may play in inflammasome activation, is still unclear. In this study, we revealed that the purinergic receptor P2X_4_ is assembled with the receptor P2X_7_ and its associated pore, pannexin-1. ATP induces ROS production through a complex consisting of the P2X_4_, P2X_7,_ and pannexin-1. P2X_7_−mediated ROS production can activate the NLRP3 inflammasome and caspase-1. Furthermore, separate depletion or inhibition of P2X_4_, P2X_7_, or pannexin-1 complex blocks IL-1β secretion in *P. gingivalis*-infected GEC following ATP treatment. However, activation via P2X_4_ alone induces ROS generation but not inflammasome activation. These results suggest that ROS is generated through stimulation of a P2X_4_/P2X_7_/pannexin-1 complex, and reveal an unexpected role for P2X_4_, which acts as a positive regulator of inflammasome activation during microbial infection.

## Introduction

Innate immunity is the first line of defense used by the host against microbial infection. In human tissues, epithelial cells play a major role in innate immunity. Epithelial cells can not only form physical barriers, but also secrete inflammatory cytokines and chemokines in response to infection following recognition of microbial products by pattern-recognition receptors (PRRs), such as Toll-like receptors (TLR) and Nod-like receptors (NLR) [1]–[4].

The proinflammatory cytokines IL-1β and IL-18 have been linked to atherosclerosis, systemic inflammatory diseases, and autoimmune disease. Their expression and secretion are stringently controlled by pathogen-associated molecular patterns (PAMPs) and danger signals [5]–[7]. Some PAMPs like lipopolysaccharide (LPS), peptidoglycan, lipoteichoic acid, flagellin, and microbial nucleic acids induce pro-IL-1β and pro-IL-18 expression and intracellular accumulation [8], [9]. However, the maturation and secretion of these cytokines requires a danger signal like ATP or uric acid crystal, which comes from stressed or infected cell and leads to the activation of inflammasomes [10]–[13].

Inflammasomes are large multiprotein complexes that act as a caspase-1-activating platform for IL-1β and IL-18 maturation. They can be categorized by the composition of their integral PRR family member, which acts as a scaffold protein that contributes to caspase-1 recruitment, clustering, and auto-activation [12]–[17]. The best characterized inflammasome is the NLRP3 inflammasome. It contains NLRP3 as a scaffold protein, an apoptosis-associated speck-like protein containing a caspase recruitment domain (ASC) adaptor, and caspase-1 [18], [19]. Danger-associated molecular patterns (DAMPs), such as extracellular ATP, can activate the NLRP3 inflammasome through ATP-gated P2X_7_ ion channels [11], [12], [20]. Upon ATP stimulation, the P2X_7_ receptor opens a cation channel, which permits K^+^ efflux, and gradually forms a larger pore on the membrane by recruiting the hemichannel pannexin-1 to activate the NLRP3 inflammasome [21]–[23]. Although P2X_4_ is also an ATP-gated ion channel, it has not been previously described to participate in ATP-mediated caspase-1 activation.

Several downstream mechanisms have been proposed to induce NLRP3 inflammasome activation, including reactive oxygen species (ROS) production, lysosomal destabilization, K^+^ efflux, and apoptosis [12], [24]–[26]. In particular, ATP stimulation of cells has been shown to induce caspase-1 activation following ROS production, and treatment with the P2X_7_ antagonist, oxATP, attenuates ATP-induced ROS generation [12], [27]–[32]. In addition to P2X_7_ agonists, agonists of other purinergic receptors also promote ROS generation, implying that other purinergic receptors may also contribute to ATP-induced ROS production [31], [33], [34]. However, until now, no other purinergic receptor has been implicated in ATP-induced activation of the NLRP3 inflammasome other than P2X_7_.

Gingival epithelial cells (GEC) represent the first barrier to infection and are a prominent component of the innate immune system in the oral cavity. The GEC are also targets of infection, and can be infected by common periodontopathogens such as *Porphyromonas gingivalis*, *Tannerella forsythia*, and *Actinobacillus actinomycetemcomitans* [35]–[38]. Previously, we showed that *P. gingivalis*-infected GEC overexpress pro-IL-1β, but secretion of the cytokine requires a second stimulus, such as treatment with exogenous ATP, to activate caspase-1 through the NLRP3 inflammasome [34], [39]. Characterizing the cell signaling events activated by pathogens in GEC provides potential candidates to control inflammatory responses associated with periodontal disease. However, the molecular mechanisms by which the GEC respond to bacterial infections remain to be elucidated. Thus, we here investigate which purinergic receptors contribute to ATP-induced ROS production and inflammasome activation in GEC, and reveal an unexpected modulatory role for P2X_4_.

## Materials and Methods

### Cells and Chemical Reagents

*Porphyromonas gingivalis* ATCC 33277 was cultured anaerobically for 24 h at 37°C in trypticase soy broth (TSB) supplemented with yeast extract (1 mg/ml), hemin (5 µg/ml) and menadione (1 µg/ml) and used for infection as described [34].

The human immortalized gingival keratinocyte (HIGK) cell line [40], was obtained as previously described [40], [41]. Cells were cultured in serum-free defined keratinocyte-SFM (Gibco) at 37°C in a humidified incubator containing 5% CO_2_.

Primary GEC were obtained after oral surgery from healthy gingival tissue as previously described [42]. Cells were cultured as monolayers in serum-free keratinocyte growth medium (KGM) (Lonza) at 37°C in 5% CO_2_. Primary GEC were used for experimentation at ∼75–80% confluence and cultured for 24 h or 48 h before infection with *P. gingivalis* at a multiplicity of infection (M.O.I.) of 100 [34].

ATP, ADP, UTP, oxATP, PPADS, and probenecid were from Sigma-Aldrich. AMP was from Santa Cruz Biotech. 5-BDBD was from Tocris Bioscience. All primers were purchased from Fisher Scientific. Antibodies against P2X_4_ (APR-002) and P2X_7_ (APR-008) were obtained from Alomone Labs.

### RNA Extraction, Reverse Transcription PCR, and Quantitative PCR

Total RNA was isolated from 10^6^ HIGK cells using RNeasy Mini kit (Qiagen) according to the manufacturer’s protocol. cDNA was amplified from 2 µg RNA by random hexamers using TagMan Reverse Transcription Reagents kit (Applied Biosystems). The following primers were used in PCR: 5′-CGCCTTCCTCTTCGAGTATGA-3′ and 5′-AGATAACGCCCACCTTCTTATTACG-3′ for P2X_1_; 5′-GCCTACGGGATCCGCATT-3′ and 5′-TGGTGGGAATCAGGCTGAAC-3′ for P2X_2_; 5′-GCTGGACCATCGGGATCA-3′ and 5′-GAAAACCCACCCTACAAAGTAGGA-3′ for P2X_3_; 5′-CCTCTGCTTGCCCAGGTACTC-3′ and 5′-CCAGGAGATACGTTGTGCTCAA-3′ for P2X_4_; 5′-CTGCCTGTCGCTGTTCGA-3′ and 5′-GCAGGCCCACCTTCTTGTT-3′ for P2X_5_; 5′-AGGCCAGTGTGTGGTGTTCA-3′ and 5′-TCTCCACTGGGCACCAACTC-3′ for P2X_6_; 5′- TCTTCGTGATGACAAACTTTCTCAA-3′ and 5′-GTCCTGCGGGTGGGATACT-3′ for P2X_7_; and 5′-GGTGAGACAAGACCCAGAGC-3′ and 5′-GGCATCGGACCTTACACCTA-3′ for pannexin1.

The PCR cycling protocol for all primers was 94°C at 5 s, 55°C at 5 s and 68°C at 15 s. The protocol was repeated for 40 cycles and included an initial 5 min enzyme activation step at 94°C and a final 10 min extension step at 72°C. PCR products were separated by electrophoresis on a 2% agarose gel and visualized by ethidium bromide staining.

Quantitative PCR (qPCR) was carried out with 1/50 of the cDNA preparation in the Mx3000P (Stratagene) in 25 µl final volumes with the Brilliant QPCR Master Mix (Stratagene). cDNA was amplified using 200 nM of each specific sense and antisense primers. Quantitative PCR was conducted at 95°C for 10 min, followed by 40 cycles at 95°C for 30 s, 55°C for 1 min and 72°C for 30 s. The expression levels of P2X_4_, P2X_7_, and pannexin-1 were normalized to GAPDH by the comparative cycle threshold method, as described by the manufacturer (Stratagene). The primers for the genes coding P2X_4_, P2X_7_, and pannexin-1 were as above. For GAPDH, the primers were: 5′-TTAAAAGCAGCCCTGGTGAC-3′ and 5′-CTCTGCTCCTCCTGTTCGAC-3′.

### Lentiviral Infection of HIGK Cells

Immortalized GEC (HIGK) stably expressing shRNA against P2X_4_ (TRCN0000044960 and TRCN0000044962), P2X_7_ (TRCN0000045095 and TRCN0000045097), and pannexin-1 (TRCN0000156046 and TRCN0000155348) were generated by transducing the cells with lentiviral particles purchased from Sigma-Aldrich. Transduction was performed following the manufacturer’s instructions. Nontarget shRNA control cells were also generated using an irrelevant sequence (SHC002V, Sigma). Briefly, GEC were plated at 70% confluency 24 h prior to transduction, and the corresponding lentiviral transduction particles were added at M.O.I. of 3 overnight. Fresh media was added the next day, and stably infected cells were selected by addition of media containing 5 µg/ml puromycin (Sigma-Aldrich).

### Transient RNA Depletion with Sirna in Primary GEC

Expression of P2X_4_ and P2X_7_ in primary GEC was repressed with different siRNA sequences as previously described [43]. The siRNA sequences were: 5′-GCUUUCAACGGGUCUGUCATT-3′ and 5′-UGACAGACCCGUUGAAAGCTA-3′ for P2X_4_ (Ambion, LifeCell Technologies, S9957, Cat. #: 4392420); and 5′-ACAAUGUUGAGAAACGGACUCUGAT-3′ for P2X_7_ (27 mer siRNA duplexes OriGene Technologies, Cat. #: SR303325). Briefly, cells were treated with siRNA using Glycofect Transfection Reagent (Kerafast) mixed with 10 nM siRNA (stock concentration of siRNA was 20 nM) in a total volume of 100 µl. Four hours later, new cell-medium was added to the cells without removal of the transfection mixture, and cells were incubated for 36 hours. qPCR was performed to confirm the knockdown efficiency and specificity, as previously shown [44].

### ROS Measurement

ROS measurement was assayed with the ROS indicator dyes, CM-H_2_−DCFDA DCF and MitoSOX (Invitrogen), as described previously [43], [45]. In brief, cells were loaded with 2.5 µM DCF or 5 µM MitoSOX in PBS for 30 min at 37°C, washed with PBS, and treated with 100 µM or 3 mM ATP for 1 h at 37°C. Cells were counter-stained with Hoechst33342 in order to reveal the nucleus. Finally, the cells were observed by wide-field fluorescence microscope (Leica, Deerfield, IL).

### Measurement of Caspase-1 Activation by ELISA

GEC were treated with 100 µM or 3 mM ATP for 3 h and supernatants were collected and subjected to human caspase-1 immunoassay (R&D) according to manufacturer’s instructions. In brief, the caspase-1 ELISA uses monoclonal and polyclonal antibodies specific for the caspase-1 p20 subunit as capture and detection antibodies, respectively. One hundred µl of supernatant were first mixed with 50 µl of RD1W buffer and loaded onto caspase-1 monoclonal antibody coated-wells for 1.5 hrs. One hundred µl of caspase-1 antiserum was then used as detection antibodies. Anti-rabbit IgG-HRP conjugate was used for quantification. Activated caspase-1 was measured using a plate reader at 450 nm with wavelength correction at 540 nm.

### Measurement of IL-1β Secretion by ELISA

Secretion of IL-1β was measured using a commercial cytokine ELISA kit (BD Biosciences Pharmingen) as described [39].

### Co-Immunoprecipitation of Purinergic Receptors

Co-immunoprecipitation was performed with Dynabeads (Invitrogen) according to the manufacturer’s instructions. Cells were lysed with the extraction buffer, and cell extracts were incubated for 3 h at 4°C with beads pre-coupled overnight with P2X_4_ antibody. Precipitates were washed with extraction buffer and LWB with the use of a magnet and were subjected to 2X sample buffer and heated to 99°C for 10 min. The eluted proteins were analyzed by Western blot as previously described [46].

## Results

### ATP Induces ROS Generation in GEC

**Figure 1:**
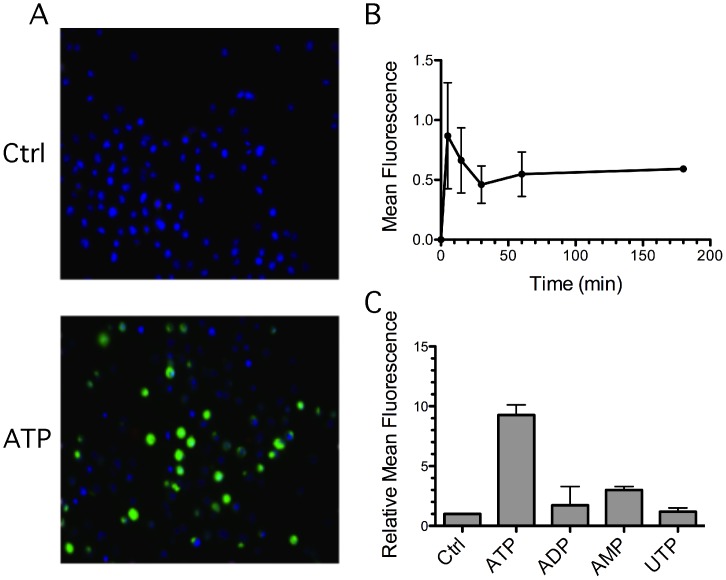
ROS production in response to ATP in GEC. (A) Immortalized GEC (HIGK) were treated for 1 hour with 3 mM ATP or left untreated as a control and stained with DCF (green) to detect ROS by fluorescence microscopy. Hoechst (blue) was used to stain nuclei. (B) ROS generation was measured with DCF at the indicated time points after 3 mM ATP stimulation. The mean fluorescence was quantified by image J and normalized for the number of cells. (C) Quantification of ROS production induced by different nucleotides. GEC were treated with 3 mM of ATP, ADP, AMP, or UTP, or left untreated for 1 hour, and analyzed by image J as in (B). The bars show the average values and SD of three independent experiments.

It has been shown stimulation with ATP results in high levels of ROS generated in alveolar macrophages and primary GEC [27], [34]. In order to characterize ATP-induced ROS production in GEC, we used a stable GEC cell line, the human immortalized gingival keratinocyte cell line (HIGK) [40], stained with carboxy-H_2_DCFDA (DCF), which remains nonfluorescent until its deacetylation and oxidation. Fluorescence microscopy images showed a significant increase of DCF fluorescence in 3 mM ATP stimulated HIGK cells (Figure 1A). Quantitative analysis of fluorescence microscopy data showed that the fluorescence in ATP-treated cells was about 9 times higher than in cells without treatment. Furthermore, the cells responded quickly to ATP stimulation within 5 minutes and reached a steady state from 30 minutes to at least 3 hours (Figure 1, B and C). Treatment with other extracellular nucleotides such as ADP, AMP, or UTP was unable to induce significant ROS generation in the cells (Figure 1C). These results suggest that stimulation with ATP, unlike other nucleotides, can induce ROS production in GECs.

### Synergistic Effects of NADPH Oxidase and Mitochondria During ROS Generation

**Figure 2:**
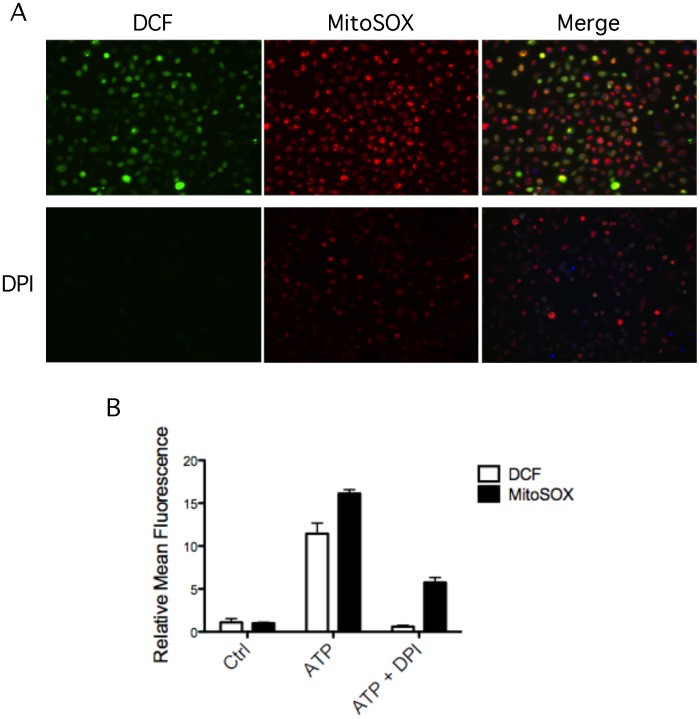
Synergistic effects of NADPH oxidase and mitochondria in ROS production. (A) HIGK were pretreated with 50 µM DPI for 30 minutes followed by 3 mM ATP stimulation, and stained with DCF (green) or MitoSOX (red) to detect ROS by fluorescence microscopy. (B) The fluorescence was quantified in three independent experiments as in Figure 1.

Two possible sources of ROS that activate the inflammassome have been described. One may be due to activation of nicotinamide adenine dinucleotide phosphate (NADPH) oxidase, triggered by frustrated phagocytosis [47]. The other may due to the main intracellular source of ROS, the mitochondria [30]. Recent studies have uncovered the existence of cross-talk between the NADPH oxidase and mitochondria [48]. In GECs, it was demonstrated that ATP induces both cytosolic and mROS production (Choi, 2013). To verify whether NADPH oxidase-induced ROS by ATP may modulate mROS production in GECs, we measured ATP-induced mROS generation after inhibiting NADPH oxidase with diphenyleneiodonium chloride (DPI), which was previously shown to block caspase-1 activation [49]. As shown in Figure 2A and B, DPI profoundly attenuated ATP-triggered cytosolic ROS production, as detected by DCF, confirming that ATP treatment induces ROS through NADPH oxidase. To examine if NADPH oxidase could also modulate ATP-induced mROS production, we used MitoSOX to detect superoxide in mitochondria. Quantitative analysis of fluorescence micrographs confirmed that ATP treatment also triggers mROS production, in agreement with previous results [45]. The increase of mROS can be partially inhibited by DPI, implying that NADPH oxidase plays a significant role in mROS generation (Figure 2, A and B). Taken together, these results suggest that ATP-induced NADPH activation can synergistically promote mROS production in GECs.

### Purinergic Receptors Involved in ATP-induced ROS Generation

**Figure 3:**
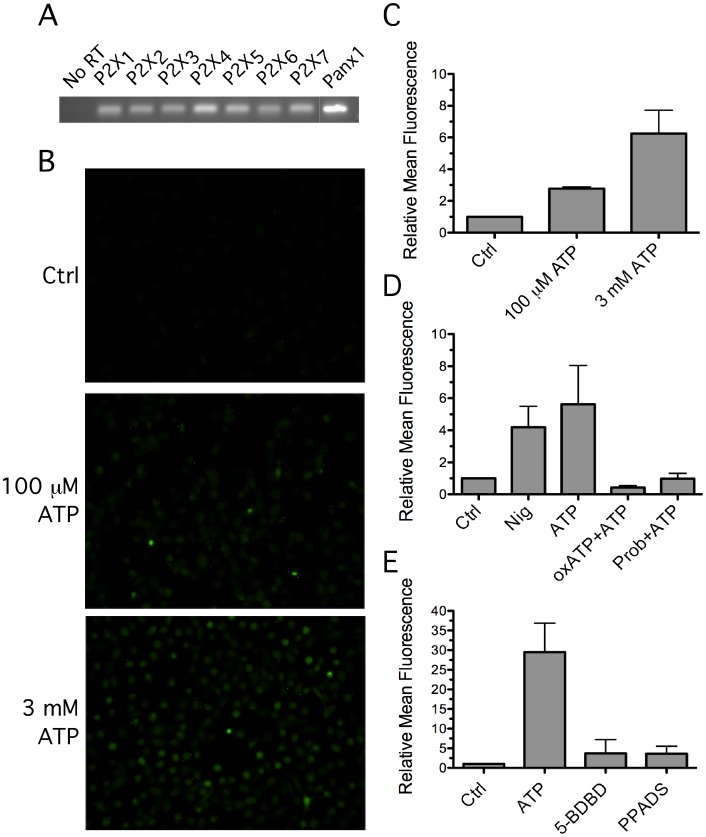
Involvement of P2X_4_, P2X_7_, and pannexin-1 in ATP-mediated ROS generation. (**A**) Total RNA from immortalized GEC was extracted and reversed transcribed (RT) to cDNA. The cDNA was used to perform PCR with the primers specific for the indicated genes, and the PCR products were finally visualized by EtBr staining. (**B**) GEC were treated with 100 µM or 3 mM ATP as indicated for 1 hour, and ROS production was measured with DCF staining and visualized by fluorescence microcopy. Quantification of the fluorescence in three independent experiments with SD is shown in (**C**). (D–E) ROS production was measured by DCF staining in GEC stimulated under different conditions. GEC were left untreated or stimulated with 20 µM nigericin or 3 mM ATP for 1 hour followed by fluorescence microscopy. Diminished ROS generation by different receptor antagonists was examined by pretreating cells with 100 µM oxATP for 30 minutes, 1 mM probenecid for 10 minutes, or 50 µM 5-BDBD or 100 µM PPADS for 15 minutes, followed by 3 mM ATP stimulation for 1 hour.

It has been proposed that extracellular ATP induces ROS production through ligation of the ATP-gated P2X_7_ ion channel, in association with the pore-forming hemi-channel, pannexin-1, in macrophages and neurons [19], [21], [50]. To investigate whether ATP-induced ROS production in GEC takes place through P2X_7_, we examined the gene expression levels of purinergic receptors in HIGK cells. In agreement with our previous description of primary GEC [51], the HIGK cells express P2X_1_ throuh P2X_7_. In addition, the HIGK cells express pannexin-1 (Figure3A).

P2X- and pannexin-1-dependent responses in HIGK cells were next examined by fluorescence microscopy (Figure 3B). Consistent with previous results, 3 mM ATP stimulated a large level of ROS production, suggesting that ATP mediates ROS production through P2X_7_ ligation (Figure 3, B and C). A role for P2X_7_ was further confirmed by showing that ATP-induced ROS production was inhibited by pretreatment with the P2X_7_ and pannexin-1 antagonists, oxATP and probenecid, respectively (Figure 3D). Moreover, as illustrated in Figure 3E, treatment with the selective P2X_7_ antagonist, PPADS, significantly blocked ATP-induced ROS generation. These data suggest that P2X_7_ may be involved in ATP-induced ROS generation in GEC.

Unexpectedly, treatment with 100 µM ATP also elicited ROS generation but to a lower extent than with 3 mM ATP (Figure 3, A and B). P2X_4_ is considered to mediate high affinity responses to ATP stimulation, at lower concentrations than for P2X_7_ [31], [33], [52], [53]. Thus, these results suggested that P2X_4_ may be involved in ATP-mediated ROS production. To test this possibility, we pretreated cells with the potent P2X_4_ antagonist, 5-BDBD. As shown in Figure 3E, pretreatment with 5-BDBD significantly blocked ATP-induced ROS generation. Taken together, these results suggest that ATP may elicit ROS generation through P2X_4_ and P2X_7_ ligation in GEC.

### Confirmation by RNA Interference for Role of P2X_7_, P2X_4_ and Pannexin-1 in ATP-Mediated ROS Production

**Figure 4:**
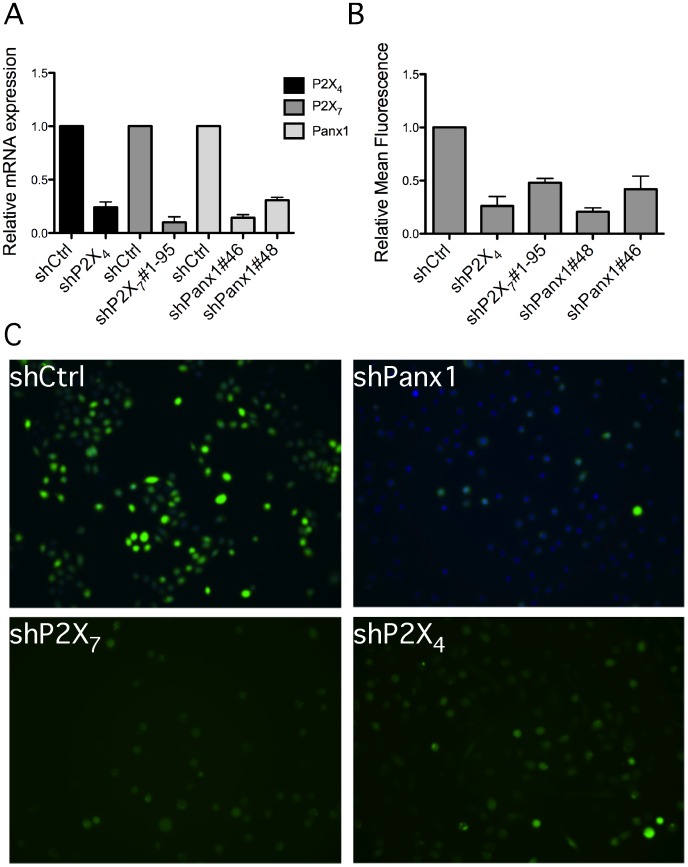
Diminished ATP-induced ROS production due to depletion of P2X_4_ or P2X_7_ by RNA interference. (**A**) Immortalized GEC were transduced with lentiviruses carrying the indicated shRNA-expressing plasmid for 1 day and selected with puromycin. After selection, cells were collected and total RNA was analyzed by qPCR to confirm knockdown efficiency. (**B**) DCF staining of ROS production after 3 mM ATP stimulation for 1 hour in different cell lines. The fluorescence shown in (**C**) was quantified as in Figure 1 and normalized to shCtrl, which was transduced with control, non-mammalian shRNA.

As inhibitor studies suggested that P2X_4_ may be involved in ATP-dependent ROS responses, we examined this unexpected result by stably depleting P2X_4_, P2X_7_ and pannexin-1 by lentiviral delivery of specific shRNA. Specific cepletion efficiency in each cell line was validated individually by qPCR, as we have previously done to show specific depletion of purinergic receptors by RNA interference [44]. As shown in Figure 4A, the mRNA levels of P2X_4_, P2X_7_, and pannexin-1 were reduced by at least ∼70% in comparison to cells transduced with control shRNA virus particles. Depletion was specific, as P2X_4_ depletion did not affect P2X_7_ expression, and conversely, P2X_7_ depletion did not affect P2X_4_ expression (not shown). In agreement with Figure 3, depletion of P2X_7_ or pannexin-1 resulted in attenuation of ROS production after ATP stimulation, compared to GEC transduced with control shRNA. Although depletion of P2X_4_ by RNA interference was less efficient than for P2X_7_, P2X_4_ depletion resulted in a dramatic decrease in ATP-mediated ROS production (Figure 4, B and C). Collectively, these findings indicate that both P2X_4_ and P2X_7_ contribute to ROS generation after ATP treatment of GEC.

### ATP Ligation by P2X_4_/P2X_7_/Pannexin-1 Complex Leads to Inflammasome Activation in GEC

**Figure 5:**
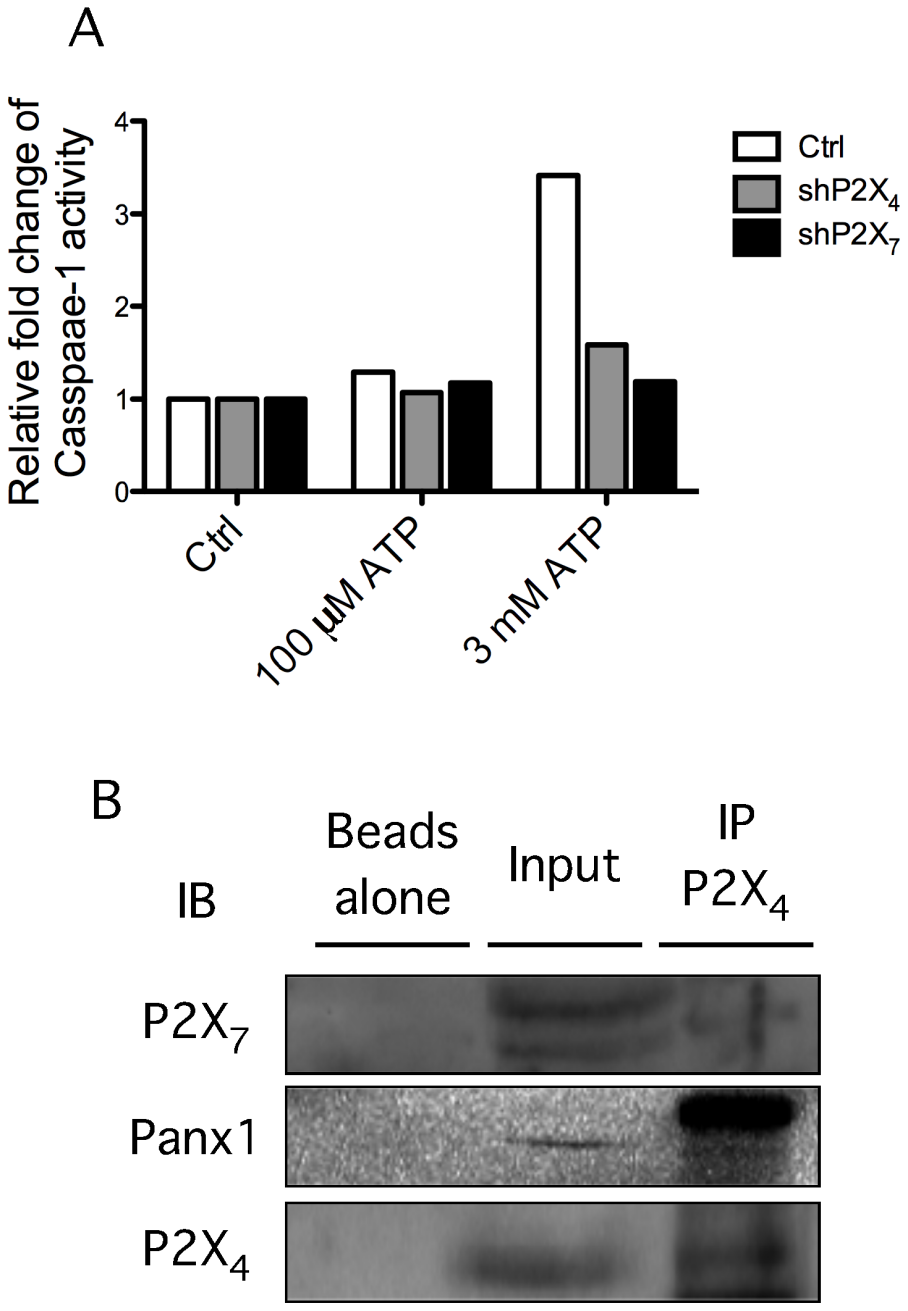
Impaired ATP-stimulated caspase-1 activation due to disruption of the complex containing P2X_4_, P2X_7_, and pannexin-1. (A) Immortalized GEC that had been depleted of P2X_4_ or P2X_7_ using lentiviral particles were treated with 100 µM or 3 mM ATP for 3 hours, and supernatants were collected for caspase-1 activity measurement. Caspase-1 activity was measured by ELISA as described in Material and Methods. (B) Total proteins isolated from GEC were subjected to immunoprecipitation (IP) by a polyclonal anti-P2X_4_ antibody or Dynabeads as a control. Precipitates or total protein extract (as input) were resolved on SDS-PAGE and analyzed on immunoblots with anti-P2X_7_ (top), anti-pannexin-1 (middle), or anti-P2X_4_ (bottom) antibodies.

We previously showed that ATP treatment of GEC leads to NLRP3 inflammasome activation [39]. As ROS production has been associated with inflammasome and caspase-1 activation [12], [27], [29], [30], we evaluated whether ATP-mediated caspase-1 activation in GEC takes place through P2X_4_/P2X_7_ ligation. Using ELISA to measure secretion of activated caspase-1, we observed that treatment of GEC with 100 µM ATP was insufficient for caspase-1 activation, even though ROS generation was induced. In contrast, 3 mM ATP treatment resulted in high levels of caspase-1 activation in GEC stably-expressing the control shRNA (Figure 5A); but the activation of caspase-1 by 3 mM ATP treatment was abrogated when either P2X_4_ or P2X_7_ were depleted in GEC (Figure 5A). Thus, treatment with 3 mM ATP induced ROS production via the P2X_4_/P2X_7_ complex and activated the NLRP3 inflammasome. However, 100 µM ATP stimulation induced ROS generation through P2X_4_ ligation, but stimulation with this concentration of ATP was not sufficient to activate the inflammasome.

The non-redundant roles of P2X_4_ and P2X_7_ in ATP-induced ROS generation led us to hypothesize that P2X_4_ and P2X_7_ may be associated in the membrane and function as a physical complex in ATP-mediated responses in GEC. Therefore, we examined physical associations between P2X_4_ and P2X_7_ in GEC by performing co-immunoprecipitation experiments. After precipitating endogenous P2X_4_ using an anti-P2X_4_ antibody, we observed that P2X_7_ and pannexin-1 were detected in the immunoprecipitate (Figure 5B). Taken together, these data indicate that P2X_4_, P2X_7_, and pannexin-1 form a heterocomplex in GEC, and play non-redundant roles in ATP-induced ROS generation.

### ATP Ligation of P2X_4_/P2X_7_/Pannexin-1 Contributes to Secretion of pro-inflammatory Cytokines Secretion in Primary GEC Infected with *P. gingivalis*

Previously we had reported that infection of GEC with *P. gingivalis* leads to expression of pro-IL-1β and its accumulation within the infected cell. However, secretion of IL-1β requires a second signal, such as the danger signal ATP, in order to activate the NLRP3 inflammasome and caspase-1, allowing processing and secretion of the mature IL-1β [39].

**Figure 6:**
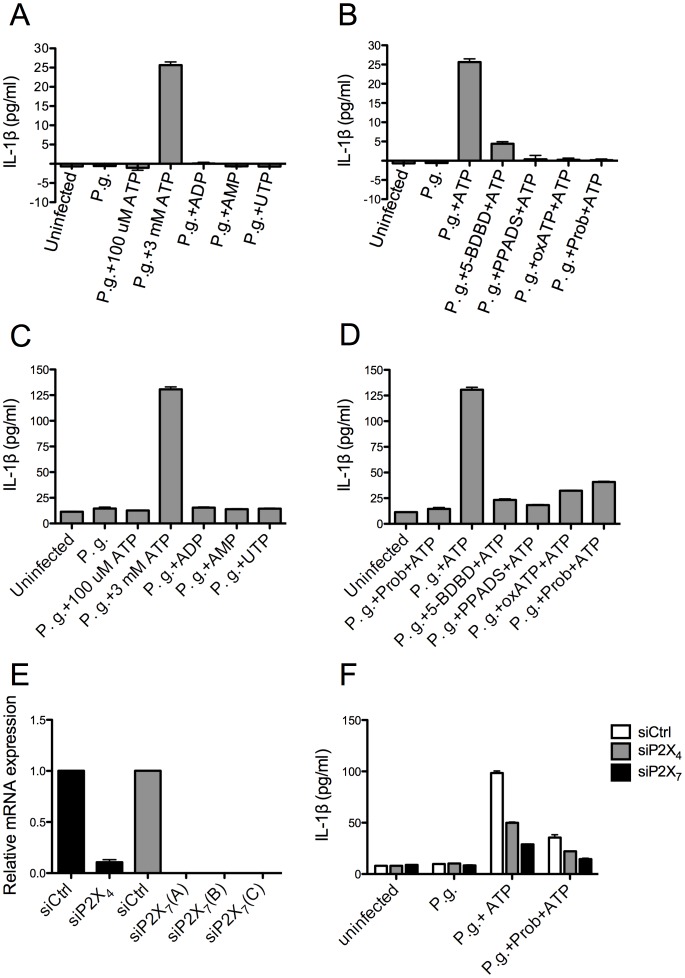
Abrogation of ATP-induced IL-1β secretion in *P.* *gingivalis*-infected GEC by inhibition of P2X_4_, P2X_7_, or pannexin-1. Primary GEC (C and D) and immortalized GEC (A and B) were infected with or without *P. gingivalis* (*P.g.*) at an M.O.I. of 100 for 6 hours, followed by treatment with different pharmaceutical agents. Infected cells were treated with 100 µM ATP, 3 mM ATP, 3 mM ADP, 3 mM AMP, or 3 mM UTP individually for 1 hour (A and C). Alternatively, infected cells were pre-treated with 50 µM 5-BDBD for 15 minutes, 100 µM PPADS for 15 minutes, 100 µM oxATP for 30 minutes, or 1 mM probenecid for 10 minutes, followed by treatment with 3 mM ATP for 1 hour (B and D). The supernatants were collected and subjected to ELISA to measure IL-1β secretion. (E) Primary GEC were transfected with siRNA sequences against P2X_4_ or P2X_7_ for one day, and mRNA levels were detected by qPCR. (F) Primary GEC depleted of P2X_4_ or P2X_7_ were infected with *P. gingivalis* (*P.g.*) and treated with probenecid and 3 mM ATP as shown in (B). IL-1β secretion in the supernatants was analyzed by ELISA. The values showed averages and SD from duplicate samples, which were obtained from three separate experiments.

Given the unexpected observation that P2X_4_ can modulate ATP-dependent caspase-1 activation in the immortalized HIGK cells, we examined whether a similar effect could be observed in immortalized (HIGK) cells and primary GEC during infection with *P. gingivalis*. As in our previous studies, neither *P. gingivalis* infection alone nor infection combined with 100 µM ATP treatment could induce IL-1β secretion by HIGK cells. Only infected cells treated with 3 mM ATP, but not other nucleotides, could promote Il-1β secretion (Figure 6A). Similarly, using primary GEC, we found that ATP, but not other nucleotides, could promote IL-1β secretion by infected cells (Figure 6C). We also consistently observed that primary GEC produce and secrete higher levels of IL-1β than HIGK cells (Figure 6).

Furthermore, pretreatment of infected HIGK or primary GEC with the P2X_7_ antagonists, PPADS and oxATP, blocked ATP-dependent IL-1β secretion. In addition, the pannexin-1 inhibitor, probenecid, also abrogated IL-1β secretion. Finally, inhibition of P2X_4_ by 5-BDBD reduced the amount of IL-1β secretion, even though the cells were treated with 3 mM ATP, which stimulates signaling via P2X_7_ (Figure 6B and D).

To further confirm a role for P2X_4_ and P2X_7_ in IL-1β, we used siRNA to deplete P2X_4_ and P2X_7_ in primary GEC individually. (In our hands, siRNA treatment is more effective than shRNA delivery for RNA interference in primary GEC.) Figure 6E showed that P2X_4_ or P2X_7_ mRNA levels were depleted with an efficiency of over 80% in primary GEC. Similarly to our previous results [39], *P. gingivalis* infection followed by 3 mM ATP treatment caused IL-1β secretion by the primary GEC that had been treated with control siRNA. However, depletion of P2X_4_ or P2X_7_ reduced significantly IL-1β secretion, which again showed a non-redundant role for P2X_4_ and P2X_7_ in ATP-dependent IL-1β secretion. Probenecid treatment prior to ATP stimulation repressed even further the IL-1β secretion in P2X_4_ and P2X_7_ knockdown cells, consistent with a role for pannexin-1 in IL-1β secretion by primary GEC. All these results imply that a P2X_4_/P2X_7_/pannexin-1 complex is required for IL-1β secretion in response to ATP treatment of *P. gingivalis*-infected cells.

## Discussion

Our results show that P2X_4_, P2X_7_, and pannexin-1 contribute to ROS generation and are associated with inflammasome activation in GEC. Consistent with this possibility, previous studies have suggested that P2X_4_ and P2X_7_ may behave as heteromeric receptors on bone marrow derived macrophages (BMDM) [54], [55]. Similarly, ATP-induced cell death of mouse macrophages was shown to involve the P2X_4_ receptor, initiating Ca^2+^ influx upon stimulation with ATP and contributing to pore formation by activation of the P2X_7_ receptor [56], [57]. These findings suggest the functionality and dependence of the P2X_4_ and P2X_7_ receptors on each other.

In GEC, we found that extracellular nucleotide-induced ROS production occurred within a few minutes and was specific for ATP stimulation. We then characterized expression of possible target receptors and tested whether specific inhibitors for these receptors could block ROS generation. Inhibitors of P2X_4_, P2X_7_, and pannexin-1 reduced significantly ATP-dependent production of ROS. To further evaluate the functionality of the receptors, we depleted either purinergic receptor or pannexin-1 by RNA interference, and find that both purinergic receptors and pannexin-1 are required for efficient ATP-induced ROS production in primary or immortalized GEC. Our findings differ from another study, which showed that depletion of the P2X_4_ receptor increased ATP-mediated ROS production in the macrophage cell line, RAW264.7 cells [56], [57]. The conflicting results may be attributed to different cells lines, but we also used primary GEC and found similar results as with the HIGK cells.

It has been proposed that either DAMPs or PAMPs could trigger ROS production, which leads to NLRP3 inflammasome activation [19]. However, the intracellular origin of ROS remains debated. Previous studies demonstrated that inhibiting NADPH oxidases with pharmacological inhibitors such as DPI or depletion by siRNA significantly decreased caspase-1 activity and IL-1β maturation in macrophages stimulated with DAMPs or PAMPS, indicating that NADPH oxidase-elicited ROS play a role in inflammasome activation [47], [58]–[60]. Subsequently, another intracellular source of ROS, mitochondria, was also reported to activate NLRP3 in response to DAMPs or PAMPs by inducing oxidation and release of mitochondrial DNA [30], [61], [62]. In GECs, a recent study demonstrated that ATP stimulation results in NADPH-induced ROS generation via P2X_7_ ligation which also promotes mROS generation, indicating that NADPH oxidase and mitochondria produce ROS synergistically [45]. Consistent with these findings, we showed that inhibition of NADPH oxidiase also decreased oATP-induced mROS generation.

Our studies show that that pannexin-1 is indispensable for ATP-induced NLRP3 activation in GECs. However, recent genetic evidence showed normal NLRP3 inflammasome function in macrophages derived from *Panx1*-deficient mice [63], [64]. This discrepancy may be explained by assuming that pannexin-1 plays a different role in different cell types. For example, in neurons, pannexin-1 is involved in inflammasome-induced cell death, as shown through the use of pannexin-1 depletion and *Panx1*-deficient mice [65]–[67].

We have previously reported that treatment of GEC with ATP concentrations that stimulate P2X_7_ leads to activation of the inflammasome and caspase-1 [39]. However, we now find that depletion of either P2X_4_ or P2X_7_ results in decreased caspase-1 activation in GEC. ROS is produced when either P2X_4_ or P2X_7_ are stimulated, but caspase-1 is activated only when GEC are treated with ATP concentrations that activate P2X_7_. Similarly, IL-1β secretion from *P. gingivalis*-infected cells, which requires caspase-1 activation, could be induced by treatment of the infected cells with ATP concentrations that stimulate P2X_7_, but inhibiting or depleting either P2X_4_ or P2X_7_ resulted in significantly lower levels of IL-1β secretion.

**Figure 7:**
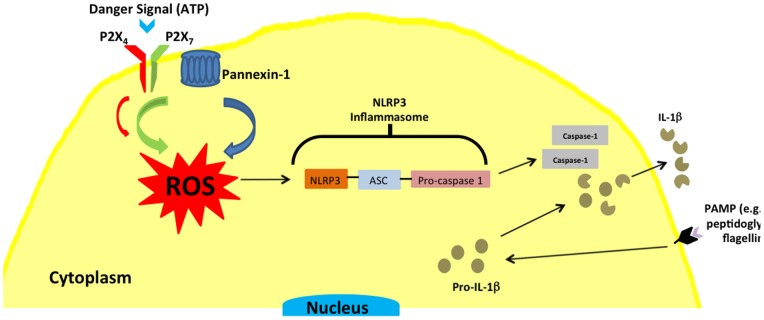
Model showing the role of P2X_4_ and P2X_7_ in ROS production and inflammasome activation in GEC stimulated with extracellular ATP.

Taken together, these results suggested that P2X_4_ stimulation may not be sufficient for activation of caspase-1, but P2X_4_ may form a complex with P2X_7_, which could explain why P2X_4_ depletion results in loss of P2X_7_-mediated signaling. We confirmed this hypothesis by demonstrating by co-immunprecipitation experiments that P2X_4_ is physically associated with P2X_7_ and pannexin-1 in GEC. P2X_4_ and P2X_7_ have previously been shown to also form heteromeric receptors in BMDM [54]. Thus, these results suggest that P2X_7_ stimulation is required for caspase-1 activation, but P2X_4_, through its presence in the P2X_4_/P2X_7_/pannexin-1 complex, modulates the activity of P2X_7_ (Figure 7).

Here, we provide an initial insight into how signaling through P2X_4_, P2X_7_, and pannexin-1 may activate caspase-1 in GEC. The same complex is involved in secretion of IL-1β from GEC that had been primed by *P. gingivalis* infection. Thus, understanding the triggers for P2X_7_−dependent ROS generation and caspase-1 activation could aid in drug discovery and development of therapeutic approaches for diseases associated with *P. gingivalis*, such as periodontal disease and cardiovascular disease.

An obvious question is the intracellular source of ROS in GEC following P2X4 or P2X7 stimulation, which could be from mitochondria and/or the NADPH oxidase on the plasma membrane [34]. A larger challenge may be to identify the molecular mechanisms that allow caspase-1 to be activated only after P2X_7_ stimulation, even though both P2X_4_ and P2X_7_ ligation leads to ROS production.

## References

[1] Hornef MW, Bogdan C (2005) The role of epithelial Toll-like receptor expression in host defense and microbial tolerance. Journal of endotoxin research 11: 124–128. 15949140 10.1179/096805105X35224

[2] Viala J, Chaput C, Boneca IG, Cardona A, Girardin SE, et al (2004) Nod1 responds to peptidoglycan delivered by the Helicobacter pylori cag pathogenicity island. Nature immunology 5: 1166–1174. 15489856 10.1038/ni1131

[3] Chamaillard M, Inohara N, Nunez G (2004) Battling enteroinvasive bacteria: Nod1 comes to the rescue. Trends in microbiology 12: 529–532. 15539110 10.1016/j.tim.2004.10.001

[4] Philpott DJ, Girardin SE, Sansonetti PJ (2001) Innate immune responses of epithelial cells following infection with bacterial pathogens. Current opinion in immunology 13: 410–416. 11498296 10.1016/s0952-7915(00)00235-1

[5] Gerdes N, Sukhova GK, Libby P, Reynolds RS, Young JL, et al (2002) Expression of interleukin (IL)-18 and functional IL-18 receptor on human vascular endothelial cells, smooth muscle cells, and macrophages: implications for atherogenesis. The Journal of experimental medicine 195: 245–257. 11805151 10.1084/jem.20011022 PMC2193607

[6] Dinarello CA (2005) Blocking IL-1 in systemic inflammation. The Journal of experimental medicine 201: 1355–1359. 15867089 10.1084/jem.20050640 PMC2213199

[7] Dinarello CA (2006) Interleukin 1 and interleukin 18 as mediators of inflammation and the aging process. The American journal of clinical nutrition 83: 447S–455S. 16470011 10.1093/ajcn/83.2.447S

[8] Ishii KJ, Koyama S, Nakagawa A, Coban C, Akira S (2008) Host innate immune receptors and beyond: making sense of microbial infections. Cell host & microbe 3: 352–363. 18541212 10.1016/j.chom.2008.05.003

[9] Franchi L, Eigenbrod T, Munoz-Planillo R, Nunez G (2009) The inflammasome: a caspase-1-activation platform that regulates immune responses and disease pathogenesis. Nature immunology 10: 241–247. 19221555 10.1038/ni.1703 PMC2820724

[10] Zitvogel L, Kepp O, Galluzzi L, Kroemer G (2012) Inflammasomes in carcinogenesis and anticancer immune responses. Nature immunology 13: 343–351. 22430787 10.1038/ni.2224

[11] Lich JD, Arthur JC, Ting JP (2006) Cryopyrin: in from the cold. Immunity 24: 241–243. 16546091 10.1016/j.immuni.2006.03.004

[12] Said-Sadier N, Ojcius DM (2012) Alarmins, inflammasomes and immunity. Biomed J 35: 437–449. 23442356 10.4103/2319-4170.104408 PMC4074086

[13] Ciraci C, Janczy JR, Sutterwala FS, Cassel SL (2012) Control of innate and adaptive immunity by the inflammasome. Microbes Infect 14: 1263–1270. 22841804 10.1016/j.micinf.2012.07.007 PMC3511629

[14] Martinon F, Burns K, Tschopp J (2002) The inflammasome: a molecular platform triggering activation of inflammatory caspases and processing of proIL-beta. Molecular cell 10: 417–426. 12191486 10.1016/s1097-2765(02)00599-3

[15] Kanneganti TD (2010) Central roles of NLRs and inflammasomes in viral infection. Nature reviews Immunology 10: 688–698. 10.1038/nri2851 PMC3909537 20847744

[16] Lupfer C, Kanneganti TD (2012) The role of inflammasome modulation in virulence. Virulence 3. 10.4161/viru.20266 PMC3442838 22546900

[17] Broz P, Monack DM (2011) Molecular mechanisms of inflammasome activation during microbial infections. Immunological reviews 243: 174–190. 21884176 10.1111/j.1600-065X.2011.01041.x PMC3170129

[18] Davis BK, Wen H, Ting JP (2011) The inflammasome NLRs in immunity, inflammation, and associated diseases. Annual review of immunology 29: 707–735. 10.1146/annurev-immunol-031210-101405 PMC4067317 21219188

[19] Schroder K, Tschopp J (2010) The inflammasomes. Cell 140: 821–832. 20303873 10.1016/j.cell.2010.01.040

[20] Meylan E, Tschopp J, Karin M (2006) Intracellular pattern recognition receptors in the host response. Nature 442: 39–44. 16823444 10.1038/nature04946

[21] Kanneganti TD, Lamkanfi M, Kim YG, Chen G, Park JH, et al (2007) Pannexin-1-mediated recognition of bacterial molecules activates the cryopyrin inflammasome independent of Toll-like receptor signaling. Immunity 26: 433–443. 17433728 10.1016/j.immuni.2007.03.008

[22] Locovei S, Scemes E, Qiu F, Spray DC, Dahl G (2007) Pannexin1 is part of the pore forming unit of the P2×(7) receptor death complex. FEBS letters 581: 483–488. 17240370 10.1016/j.febslet.2006.12.056 PMC1868681

[23] Kahlenberg JM, Dubyak GR (2004) Mechanisms of caspase-1 activation by P2×7 receptor-mediated K+ release. American journal of physiology Cell physiology 286: C1100–1108. 15075209 10.1152/ajpcell.00494.2003

[24] Shi Y, Mucsi AD, Ng G (2010) Monosodium urate crystals in inflammation and immunity. Immunological reviews 233: 203–217. 20193001 10.1111/j.0105-2896.2009.00851.x

[25] Halle A, Hornung V, Petzold GC, Stewart CR, Monks BG, et al (2008) The NALP3 inflammasome is involved in the innate immune response to amyloid-beta. Nature immunology 9: 857–865. 18604209 10.1038/ni.1636 PMC3101478

[26] Hornung V, Bauernfeind F, Halle A, Samstad EO, Kono H, et al (2008) Silica crystals and aluminum salts activate the NALP3 inflammasome through phagosomal destabilization. Nature immunology 9: 847–856. 18604214 10.1038/ni.1631 PMC2834784

[27] Cruz CM, Rinna A, Forman HJ, Ventura AL, Persechini PM, et al (2007) ATP activates a reactive oxygen species-dependent oxidative stress response and secretion of proinflammatory cytokines in macrophages. The Journal of biological chemistry 282: 2871–2879. 17132626 10.1074/jbc.M608083200 PMC2693903

[28] Arlehamn CS, Petrilli V, Gross O, Tschopp J, Evans TJ (2010) The role of potassium in inflammasome activation by bacteria. The Journal of biological chemistry 285: 10508–10518. 20097760 10.1074/jbc.M109.067298 PMC2856258

[29] Zhou R, Tardivel A, Thorens B, Choi I, Tschopp J (2010) Thioredoxin-interacting protein links oxidative stress to inflammasome activation. Nature immunology 11: 136–140. 20023662 10.1038/ni.1831

[30] Zhou R, Yazdi AS, Menu P, Tschopp J (2011) A role for mitochondria in NLRP3 inflammasome activation. Nature 469: 221–225. 21124315 10.1038/nature09663

[31] Bowler JW, Bailey RJ, North RA, Surprenant A (2003) P2×4, P2Y1 and P2Y2 receptors on rat alveolar macrophages. British journal of pharmacology 140: 567–575. 12970084 10.1038/sj.bjp.0705459 PMC1574050

[32] Rayah A, Kanellopoulos JM, Di Virgilio F (2012) P2 receptors and immunity. Microbes Infect 14: 1254–1262. 22909902 10.1016/j.micinf.2012.07.006 PMC3514633

[33] North RA (2002) Molecular physiology of P2X receptors. Physiological reviews 82: 1013–1067. 12270951 10.1152/physrev.00015.2002

[34] Choi CH, Spooner R, Deguzman J, Koutouzis T, Ojcius DM, et al.. (2012) Porphyromonas gingivalis-nucleoside-diphosphate-kinase inhibits ATP-induced reactive-oxygen-species via P2×(7) receptor/NADPH-oxidase signalling and contributes to persistence. Cellular microbiology in press. 10.1111/cmi.12089 PMC3625498 23241000

[35] Lamont RJ, Yilmaz O (2002) In or out: the invasiveness of oral bacteria. Periodontology 2000 30: 61–69. 12236896 10.1034/j.1600-0757.2002.03006.x

[36] Nisapakultorn K, Ross KF, Herzberg MC (2001) Calprotectin expression in vitro by oral epithelial cells confers resistance to infection by Porphyromonas gingivalis. Infection and immunity 69: 4242–4247. 11401960 10.1128/IAI.69.7.4242-4247.2001 PMC98457

[37] Socransky SS, Haffajee AD, Cugini MA, Smith C, Kent RL Jr (1998) Microbial complexes in subgingival plaque. Journal of clinical periodontology 25: 134–144. 9495612 10.1111/j.1600-051x.1998.tb02419.x

[38] Christersson LA, Wikesjo UM, Albini B, Zambon JJ, Genco RJ (1987) Tissue localization of Actinobacillus actinomycetemcomitans in human periodontitis. II. Correlation between immunofluorescence and culture techniques. Journal of periodontology 58: 540–545. 3305857 10.1902/jop.1987.58.8.540

[39] Yilmaz O, Sater AA, Yao L, Koutouzis T, Pettengill M, et al (2010) ATP-dependent activation of an inflammasome in primary gingival epithelial cells infected by Porphyromonas gingivalis. Cellular microbiology 12: 188–198. 19811501 10.1111/j.1462-5822.2009.01390.x PMC2807919

[40] Oda D, Bigler L, Lee P, Blanton R (1996) HPV immortalization of human oral epithelial cells: a model for carcinogenesis. Exp Cell Res 226: 164–169. 8660952 10.1006/excr.1996.0215

[41] McMahon L, Schwartz K, Yilmaz O, Brown E, Ryan LK, et al (2011) Vitamin D-mediated induction of innate immunity in gingival epithelial cells. Infection and immunity 79: 2250–2256. 21422187 10.1128/IAI.00099-11 PMC3125855

[42] Yilmaz O, Jungas T, Verbeke P, Ojcius DM (2004) Activation of the phosphatidylinositol 3-kinase/Akt pathway contributes to survival of primary epithelial cells infected with the periodontal pathogen Porphyromonas gingivalis. Infection and immunity 72: 3743–3751. 15213114 10.1128/IAI.72.7.3743-3751.2004 PMC427421

[43] Abdul-Sater AA, Said-Sadier N, Lam VM, Singh B, Pettengill MA, et al (2010) Enhancement of reactive oxygen species production and chlamydial infection by the mitochondrial Nod-like family member NLRX1. The Journal of biological chemistry 285: 41637–41645. 20959452 10.1074/jbc.M110.137885 PMC3009891

[44] Séror C, Melki MT, Subra F, Raza SQ, Bras M, et al (2012) Extracellular ATP acts on P2Y2 purinergic receptors to facilitate HIV-1 infection. J Exp Med 208: 1823–1834. 10.1084/jem.20101805 PMC3171090 21859844

[45] Choi CH, Spooner R, Deguzman J, Koutouzis T, Ojcius DM, et al (2012) Porphyromonas gingivalis-nucleoside-diphosphate-kinase inhibits ATP-induced reactive-oxygen-species via P2×(7) receptor/NADPH-oxidase signalling and contributes to persistence. Cellular Microbiology 15: 961–976. 10.1111/cmi.12089 PMC3625498 23241000

[46] Huang PR, Hung SC, Wang TC (2010) Telomeric DNA-binding activities of heterogeneous nuclear ribonucleoprotein A3 in vitro and in vivo. Biochimica et biophysica acta 1803: 1164–1174. 20600361 10.1016/j.bbamcr.2010.06.003

[47] Dostert C, Petrilli V, Van Bruggen R, Steele C, Mossman BT, et al (2008) Innate immune activation through Nalp3 inflammasome sensing of asbestos and silica. Science 320: 674–677. 18403674 10.1126/science.1156995 PMC2396588

[48] Desouki MM, Kulawiec M, Bansal S, Das GM, Singh KK (2005) Cross talk between mitochondria and superoxide generating NADPH oxidase in breast and ovarian tumors. Cancer Biology & Therapy 4: 1367–1373. 16294028 10.4161/cbt.4.12.2233

[49] Cassel SL, Eisenbarth SC, Iyer SS, Sadler JJ, Colegio OR, et al (2008) The Nalp3 inflammasome is essential for the development of silicosis. Proc Natl Acad Sci USA 105: 9035–9040. 18577586 10.1073/pnas.0803933105 PMC2449360

[50] Silverman WR, de Rivero Vaccari JP, Locovei S, Qiu F, Carlsson SK, et al (2009) The pannexin 1 channel activates the inflammasome in neurons and astrocytes. The Journal of biological chemistry 284: 18143–18151. 19416975 10.1074/jbc.M109.004804 PMC2709345

[51] Yilmaz O, Yao L, Maeda K, Rose TM, Lewis EL, et al (2008) ATP scavenging by the intracellular pathogen Porphyromonas gingivalis inhibits P2×7-mediated host-cell apoptosis. Cellular microbiology 10: 863–875. 18005240 10.1111/j.1462-5822.2007.01089.x PMC2637656

[52] Negulyaev YA, Markwardt F (2000) Block by extracellular Mg2+ of single human purinergic P2×4 receptor channels expressed in human embryonic kidney cells. Neuroscience letters 279: 165–168. 10688055 10.1016/s0304-3940(99)00976-3

[53] Soto F, Garcia-Guzman M, Gomez-Hernandez JM, Hollmann M, Karschin C, et al (1996) P2X4: an ATP-activated ionotropic receptor cloned from rat brain. Proceedings of the National Academy of Sciences of the United States of America 93: 3684–3688. 8622997 10.1073/pnas.93.8.3684 PMC39672

[54] Guo C, Masin M, Qureshi OS, Murrell-Lagnado RD (2007) Evidence for functional P2×4/P2×7 heteromeric receptors. Molecular pharmacology 72: 1447–1456. 17785580 10.1124/mol.107.035980

[55] Boumechache M, Masin M, Edwardson JM, Gorecki DC, Murrell-Lagnado R (2009) Analysis of assembly and trafficking of native P2X4 and P2×7 receptor complexes in rodent immune cells. The Journal of biological chemistry 284: 13446–13454. 19304656 10.1074/jbc.M901255200 PMC2679444

[56] Kawano A, Tsukimoto M, Mori D, Noguchi T, Harada H, et al (2012) Regulation of P2×7-dependent inflammatory functions by P2×4 receptor in mouse macrophages. Biochemical and biophysical research communications 420: 102–107. 22405772 10.1016/j.bbrc.2012.02.122

[57] Kawano A, Tsukimoto M, Noguchi T, Hotta N, Harada H, et al (2012) Involvement of P2×4 receptor in P2×7 receptor-dependent cell death of mouse macrophages. Biochemical and biophysical research communications 419: 374–380. 22349510 10.1016/j.bbrc.2012.01.156

[58] Tassi S, Carta S, Vene R, Delfino L, Ciriolo MR, et al (2009) Pathogen-induced interleukin-1beta processing and secretion is regulated by a biphasic redox response. J Immunol 183: 1456–1462. 19561107 10.4049/jimmunol.0900578

[59] Meissner F, Molawi K, Zychlinsky A (2008) Superoxide dismutase 1 regulates caspase-1 and endotoxic shock. Nature Immunol 9: 866–872. 18604212 10.1038/ni.1633

[60] Carta S, Tassi S, Pettinati I, Delfino L, Dinarello CA, et al (2011) The rate of interleukin-1beta secretion in different myeloid cells varies with the extent of redox response to Toll-like receptor triggering. J Biol Chem 286: 27069–27080. 21628463 10.1074/jbc.M110.203398 PMC3149300

[61] Shimada K, Crother TR, Karlin J, Dagvadorj J, Chiba N, et al (2012) Oxidized mitochondrial DNA activates the NLRP3 inflammasome during apoptosis. Immunity 36: 401–414. 22342844 10.1016/j.immuni.2012.01.009 PMC3312986

[62] Shimada K, Crother TR, Arditi M (2012) Innate immune responses to Chlamydia pneumoniae infection: role of TLRs, NLRs, and the inflammasome. Microbes Infect 14: 1301–1307. 22985781 10.1016/j.micinf.2012.08.004 PMC3511600

[63] Qu Y, Misaghi S, Newton K, Gilmour LL, Louie S, et al (2011) Pannexin-1 is required for ATP release during apoptosis but not for inflammasome activation. J Immunol 186: 6553–6561. 21508259 10.4049/jimmunol.1100478

[64] Wang H, Xing Y, Mao L, Luo Y, Kang L, et al.. (2013) Pannexin-1 influences peritoneal cavity cell population but is not involved in NLRP3 inflammasome activation. Protein Cell. 10.1007/s13238-013-2114-1 PMC4875518 23549611

[65] Dvoriantchikova G, Ivanov D, Barakat D, Grinberg A, Wen R, et al (2012) Genetic ablation of Pannexin1 protects retinal neurons from ischemic injury. PLoS ONE 7: e31991. 22384122 10.1371/journal.pone.0031991 PMC3285635

[66] de Rivero Vaccari JP, Lotocki G, Marcillo AE, Dietrich WD, Keane RW (2008) A molecular platform in neurons regulates inflammation after spinal cord injury. J Neurosci 28: 3404–3414. 18367607 10.1523/JNEUROSCI.0157-08.2008 PMC6670583

[67] Silverman WR, de Rivero Vaccari JP, Locovei S, Qiu F, Carlsson SK, et al (2009) The pannexin 1 channel activates the inflammasome in neurons and astrocytes. J Biol Chem 284: 18143–18151. 19416975 10.1074/jbc.M109.004804 PMC2709345

